# Perceived Teacher Responses to Bullying Influence Students’ Social Cognitions

**DOI:** 10.3389/fpsyg.2020.592582

**Published:** 2020-12-01

**Authors:** Karlien Demol, Karine Verschueren, Christina Salmivalli, Hilde Colpin

**Affiliations:** ^1^School Psychology and Development in Context, Faculty of Psychology and Educational Sciences, KU Leuven, Leuven, Belgium; ^2^INVEST Research Flagship, Department of Psychology and Speech-Language Pathology, University of Turku, Turku, Finland

**Keywords:** teacher responses to bullying, student social cognitions, perceived teacher attitudes, perceived teacher moral disengagement, willingness to report bullying, bullying participant role behaviors, experimental vignettes

## Abstract

Teachers’ responses to bullying incidents are key in bullying intervention at school. Scholars have suggested that teacher responses can predict student cognitions that are associated with their bullying behaviors. However, little is known about whether and how teacher responses affect these cognitions. Therefore, the current study investigated the effects of four immediate teacher responses on four bullying-related student cognitions, using an experimental vignette design. Additionally, it was examined whether students’ own participant role behaviors in actual bullying moderated these effects. The investigated teacher responses were non-response, comforting the victim, correcting the bully, and a combination of comforting the victim and correcting the bully. The investigated student cognitions were perceived teacher attitudes toward bullying, perceived teacher moral disengagement regarding bullying, student willingness to report bullying to the teacher and student expectations regarding bullying participant role behaviors in the classroom. Fourth-to-sixth grade students (*N* = 910; 47% boys; *M*_age_ = 11.04 years, *SD* = 0.91) read a vignette describing a hypothetical teacher’s response to a same bullying incident, following random assignment to one of eight conditions (i.e., four teacher responses × two genders of bully and victim in the vignette). Afterward, students completed questionnaires about their social cognitions and manipulation checks. ANOVA demonstrated that students perceived stronger teacher anti-bullying attitudes and less teacher moral disengagement when the hypothetical teacher displayed an active response. These effects were even stronger when the teacher corrected the bully compared to when only the victim was comforted. Further, students were more willing to report bullying when the teacher corrected the bully than when the teacher only comforted the victim. Finally, students expected less pro-bullying behaviors, more defending and less victimization in the vignette’s classroom following active teacher response compared to non-response. The effects of teacher responses on student cognitions were not moderated by students’ own participant roles in bullying. Taken together, these findings emphasize the importance of active teacher responses to bullying, and especially, responses that clearly show that bullying is not tolerated. Teachers are encouraged to be aware that students can deduce beliefs from teacher responses which can, in turn, affect bullying processes in the classroom.

## Introduction

Bullying among students at school is a persistent problem predicting various difficulties for victims (Arseneault, 2018). Although prevalence rates vary, several large-scale international studies have demonstrated that bullying affects the lives of students worldwide. For instance, in the latest report of the Health Behavior in School-aged Children (HBSC) survey of the WHO, overall, 10% of youth indicated that they had been repeatedly bullied at school during the past months (Inchley et al., 2020). Bullying has mostly been defined as repeated and intentional aggressive behavior toward others who have difficulties defending themselves (Olweus, 1993). Further, it has been recognized as a group process (Salmivalli et al., 1996) embedded in social contexts such as classrooms (e.g., Saarento et al., 2015b). Victims often suffer from a wide range of health and psychosocial problems, both in the short and long run (Moore et al., 2017). Also, they have a higher risk to develop poor socioeconomic outcomes throughout the life span (e.g., Wolke and Lereya, 2015; Arseneault, 2018). Clearly, research on the risk and protective factors of bullying and victimization is critical.

Teachers play an important role in reducing bullying (Brendgen and Troop-Gordon, 2015). They are the responsible adults in class and they are expected to secure a safe learning environment and deal effectively with any negative behaviors, including bullying (Kochenderfer-Ladd and Pelletier, 2008). A limited number of mostly observational studies have reported associations between teacher responses to bullying and the levels of bullying and victimization in classrooms (e.g., Troop-Gordon and Ladd, 2015; Campaert et al., 2017). However, to date, it remains largely unclear how exactly teacher responses may impact bullying. Scholars have pointed to student cognitions as possible intervening mechanisms (Troop-Gordon and Quenette, 2010; Menesini, 2019), but, thus far, few studies have examined effects of teacher responses on student cognitions. This study addresses this gap by investigating how teacher responses to bullying affect student social cognitions, using an experimental design.

### Effects of Teacher Responses to Bullying on Students’ Social Cognitions

When teachers are confronted with bullying among their students, they can intervene in multiple ways (Campaert et al., 2017; Wachs et al., 2018). Campaert et al. (2017) distinguished five responses in their conceptualization of teacher responses, more specifically a relational and supportive response (i.e., support to victim), a confronting response (i.e., disciplinary sanction for the bully), a response that involves both the bully and victim (i.e., mediation) and the whole class (i.e., group discussion). Additionally, Campaert et al. (2017) distinguished non-response as previous research showed that sometimes teachers do not respond to bullying.

Based on socio-cognitive theory (Bandura, 1986), students are expected to cognitively process teacher responses to bullying and look for the meaning behind these responses. Consequently, students may take different cues from teacher responses which may, in turn, affect their bullying-related behaviors. Although scholars have brought attention to different bullying-related student cognitions that could be impacted by teacher responses, so far, only a small number of mostly observational studies have examined this (Troop-Gordon and Quenette, 2010; Menesini, 2019). To fill this gap, the current study experimentally investigates the effects of different teacher responses to bullying on a comprehensive set of student social cognitions that are expected to be explanatory mechanisms in the association between teacher responses and bullying. These cognitions comprise (1) perceived teacher attitudes toward bullying, (2) perceived teacher moral disengagement regarding bullying, (3) students’ willingness to report bullying to the teacher, and (4) students’ expectations regarding classmates’ participant role behaviors in bullying.

First, students may take cues regarding teacher attitudes toward bullying. By their responses to bullying, teachers can communicate what they consider appropriate and inappropriate behavior (Veenstra et al., 2014; van der Zanden et al., 2015). Based on goal-framing theory (Lindenberg, 2013), goals related with bullying behavior (e.g., achieving and maintaining high status in the peer group) are expected to be inhibited when an overarching goal to behave socially appropriately is activated. The “normative goal” that bullying is not acceptable can be activated when significant others stand for this norm. Teachers can be this significant other when students see them as authority figures who clearly stand up against bullying (Veenstra et al., 2014). Consequently, when teachers actively respond to bullying, they can promote the norm that bullying is not tolerated. Students may adjust to this norm making them less likely to bully again (Bandura, 1977; van der Zanden et al., 2015). Conversely, when teachers do not respond to bullying or only passively, they might signal that bullying is accepted which can result in more bullying (van der Zanden et al., 2015). Indeed, longitudinal studies have shown that teachers’ efforts to intervene in bullying contributed negatively to the perceived acceptability of bullying in the classroom, and lower perceived acceptability, in turn, predicted lower bullying levels (Saarento et al., 2015a). Referring to social cognitive theory (Bandura, 1986) and goal-framing theory (Veenstra et al., 2014), students are expected to perceive stronger anti-bullying attitudes in teachers who actively respond to bullying. Accordingly, weaker anti-bullying attitudes might be perceived when teachers do not respond.

Second, students may take cues of teacher moral disengagement regarding bullying. The mechanism of moral disengagement (Bandura et al., 1996) has been used to explain how people justify aggressive behavior and bullying (e.g., meta-analysis by Gini et al., 2014). A cross-sectional study indicated that teacher responses to bullying affect students’ own levels of moral disengagement regarding bullying which, in turn, predicted the level of bullying (Campaert et al., 2017). Based on their findings, Campaert et al. (2017) stated that students may be less inclined to justify bullying when they understand from reasoning on teacher responses that bullying is unacceptable harmful behavior. However, to our knowledge, no previous study has investigated whether students take cues regarding moral disengagement of teachers based on their responses. In line with perceived teacher attitudes, students might perceive less moral disengagement regarding bullying from teachers who actively respond. Conversely, when teachers do not respond, students might think that this is due to teachers justifying bullying in some way.

Third, teacher responses to bullying may affect students’ willingness to report the bullying to teachers. Studies have found that students’ willingness to report bullying is predicted by teachers’ helpfulness in resolving past bullying episodes (Aceves et al., 2010), by students’ expectations regarding how teachers would respond to bullying incidents (Cortes and Kochenderfer-Ladd, 2014) and by student-perceived teacher attitudes toward bullying (Blomqvist et al., 2020). For instance, by ignoring bullying, teachers express a lack of concern and give students the message that they cannot expect any assistance (Yoon and Bauman, 2014). Also, when teachers do not respond to bullying, less negative teacher attitudes toward bullying can be perceived (Veenstra et al., 2014). As a result, it is expected that students would be less willing to report bullying to non-responding teachers. Contrarily, it is expected that students would be more willing to report bullying to teachers who actively respond.

Fourth, teacher responses may impact students’ expectations regarding classmates’ participant role behaviors in bullying. In previous research, six participant roles that students can have in the group process of bullying have been identified: besides being victimized themselves, students may bully others, assist the bullies, reinforce the bullies’ behaviors, defend and support the victimized peers, or remain non-involved outsiders (Salmivalli et al., 1996). Such responses are affected by individual as well as contextual factors (Salmivalli et al., 1996). Based on social learning theory (Bandura, 1977), teachers are expected to be role models for students. By their responses to bullying incidents, teachers can set expectations for students’ behavior and the relational climate in class (Yoon and Bauman, 2014). For instance, when teachers do not respond after witnessing bullying, they may model insensitive and uncaring behavior (Yoon and Bauman, 2014) and students might expect fellow students to neither stand up to the bully nor support the victim. However, when teachers actively respond to bullying, teachers may model sensitive behavior (van der Zanden et al., 2015) and students might expect less pro-bullying behaviors (i.e., bullying, assisting, reinforcing) and less victimization in the classroom. In addition, students might expect more fellow students to defend victims and less fellow students to stay outsiders.

While researchers have referred to student social cognitions that could be predicted by teacher responses (e.g., Troop-Gordon and Quenette, 2010), to date, only one study has tested this experimentally (Demol et al., in revision). This vignette study in the fourth to sixth grade of elementary school investigated whether responses of an hypothetical teacher to a bullying incident affected students’ own bullying attitudes, their empathy toward victims, their ideas of the teacher’s bullying attitudes and their evaluations of teacher responses. Students’ own bullying attitudes and empathy toward victims were not impacted by teacher responses. However, students’ ideas of the teacher’s attitudes toward bullying and their evaluations of the teacher responses differed among the teacher responses. A combination of confronting the bully and supporting the victim was evaluated as the most appropriate response. Further, when the bully was confronted, students perceived more anti-bullying attitudes in the teacher compared to when the victim was supported. Students perceived the least strong anti-bullying attitudes when the teacher did not respond and non-response was also judged to be the least appropriate response.

Although there is no previous research available, individual characteristics of students can be expected to moderate the effect of teacher responses on their cognitions. For instance, students’ own typical responses in bullying situations (their participant role behaviors) may predict their perceptions of teacher responses. It is possible that for relatively higher levels of victimization and defending, the effect of teacher response is stronger. Students who are more victimized or who defend more can be expected to be more tuned to teacher responses and interpret them more strongly. As a result, their cognitions can be expected to be more strongly affected by teacher responses. For instance, teacher’s non-response may yield even less willingness to report bullying to the teacher. Conversely, teacher’s active responses may yield even more willingness to report bullying. Contrarily, for relatively higher levels of pro-bullying behaviors (i.e., bullying, assisting, and reinforcing), the effect of teacher response may be less strong as these behaviors may be related to lower caring about how teachers respond to bullying.

### Current Study

Scholars have suggested that teachers’ responses to bullying incidents may impact their students’ social cognitions related with bullying processes (Troop-Gordon and Quenette, 2010; Menesini, 2019). However, to date, (experimental) research investigating the effects of teacher responses on students’ social cognitions is very scarce. The current experimental vignette study aims at further unraveling how teacher responses can impact bullying. This study investigates the effects of four teacher responses on four student cognitions that are expected to be explanatory mechanisms in the association between teacher responses and bullying.

Inspired by the work of Campaert et al. (2017), this study focuses on four immediate teacher responses to bullying, i.e., Non-response, Comforting the Victim, Correcting the Bully, and a combination of Comforting the Victim and Correcting the Bully. Further, building on available evidence, this study focuses on four student cognitions: (1) perceived teacher attitudes toward bullying, (2) perceived teacher moral disengagement regarding bullying, (3) student willingness to report bullying to the teacher, and (4) student expectations regarding participant role behaviors in the classroom.

Building on social cognitive theory (Bandura, 1986) and goal-framing theory (Veenstra et al., 2014), it is expected that when teachers actively respond to bullying (i.e., by Comforting the Victim, Correcting the Bully, or combining Comforting the Victim and Correcting the Bully) students will perceive stronger anti-bullying attitudes and less moral disengagement in the teacher. Also, it is hypothesized that active teacher responses predict more student willingness to report bullying to the teacher. Further, students are hypothesized to expect less victimization, more defending, less pro-bullying behaviors and less outsider behaviors in the classroom following active response. Non-response is hypothesized to have a negative effect, i.e., predict less strong perceived teacher anti-bullying attitudes and more teacher moral disengagement, less student willingness to report bullying, more expected victimization, less expected defending, more expected pro-bullying behaviors and more expected outsider behaviors. Further, based on theory (Bandura, 1986; Veenstra et al., 2014) and the findings of Demol et al. (in revision), teachers are expected to show more disapproval of bullying when responses are directed to bullies. Therefore, perceived teacher anti-bullying attitudes are expected to be stronger and perceived moral disengagement to be lower when the teacher corrects the bully (with or without comforting the victim). Also, students willingness to report bullying to teachers might be higher when teachers directed responses to bullies as this more explicitly shows that they tried to stop the bullying (Veenstra et al., 2014; Blomqvist et al., 2020). Other differences between the active responses (i.e., Comforting the Victim, Correcting the Bully, and both Comforting the Victim and Correcting the Bully) will be explored as theory and research regarding this topic is very limited.

Finally, this study will explore whether and how students’ own levels of different participant role behaviors in actual bullying moderate the effect of teacher responses on their cognitions. As there is no previous research available, it is difficult to formulate hypotheses. However, it can be tentatively expected that for relatively higher levels of victimization and defending, the effect of teacher response will be stronger. Contrarily, for relatively higher levels of pro-bullying behaviors (i.e., bullying, assisting, and reinforcing), the effect of teacher response may be less strong.

## Materials and Methods

### Sample

The data of this study are part of the Teachers4Victims project that investigates the role of teachers in bullying and victimization in the fourth to sixth grade of elementary school by means of a longitudinal and experimental study. Ethical approval for the project was acquired from The Social and Societal Ethics Committee (SMEC) of KU Leuven. The current study uses data from the third wave of the longitudinal study, and the experimental study (conducted about 2 weeks after the third wave). The experimental data were collected from 910 students (55 classes, 11 elementary schools, 47% boys, *M*_age_ = 11.04 years, *SD* = 0.91, range: 9.27–13.95) with active parental consent (consent rate: 81%). Of these students, 31.2% were in grade 4, 35.7% in grade 5, and 33.1% in grade 6. Most students were born in Belgium (92.1%) and spoke Dutch at home (86.8%). The other students were born in several other countries of which most in the Netherlands, Poland, Romania, and Spain. Most other languages spoken at home were French, Arabic, English, and Turkish. Of the 910 students that participated to the experimental study, 890 students also participated to the third wave of the longitudinal study.

### Procedure

Master and doctoral students at the faculty of Psychology and Educational Sciences of KU Leuven visited the schools to collect the data. The third wave of the longitudinal study took place about 2 weeks before the experimental study (May 2019). At the beginning of both data collections, the researchers explained bullying by reading aloud a description based on definition of Olweus (1993). Students were asked to follow by simultaneously reading the description on their own copy in silence. Students could reread the description at any time during data collection. At the third wave of the longitudinal study, students individually completed questionnaires about, for instance, participant role behaviors in bullying. At the experimental study, students first read a vignette with a story about a bullying incident that only differed regarding the teacher’s response to it and the gender of the hypothetical bully and victim. A between-subjects 4x2 design (teacher response × gender bully and victim) with random assignment within classes was used (112–116 students in each condition). After reading the story, students completed questionnaires about the story. Afterward, they responded to questions for manipulation checks and questions about the credibility and recognizability of the story. At the end, a debriefing was given by the researchers. At both data collections, students could ask questions to the researchers at any time.

#### Vignettes

The vignettes, including the instructions, were similar to those developed by Demol et al. (in revision) inspired by Bauman and Del Rio (2006), with the exception that the description of the actual bullying incident was somewhat shortened. Eight versions of the vignette (four teacher responses × two gender bully and victim) were created and each participant read one vignette. The vignettes only differed regarding the teacher response (four: Non-response, Comforting Victim, Correcting Bully, and Comforting Victim and Correcting Bully) and the gender of the bully and victim (two: both either boys or girls). The bully and victim’s gender were manipulated to counteract for possible gender effects. Mixed-gender versions were not developed as more versions would result in lower power and same gender bullying occurs more frequently (Baldry, 2004). The gender of the teacher could not be deduced from the story.

The vignette started with the instruction that students had to imagine having moved to a new school. Then, it was stated that, since the beginning of the school year, one classmate from the new class had been bullied by another. Next, a bullying situation between these classmates at the playground during a break was described. The bullying met definition of Olweus (1993), and consisted of verbal, physical, and relational bullying (Bauman and Del Rio, 2006):

“Imagine that you have changed schools. You have been in a new class since the beginning of this school year. *Name victim* and *name bully* are two students from your new class. Since the beginning of the school year, *name victim* has been bullied by *name bully*. Today you see the next thing happen during playtime. *Name bully* tells *name victim* that *he/she* cannot play along with the group. This makes *name victim* sad and *he/she* gets tears in *his/her* eyes. *Name bully* sees this and says: “What a cry baby you are!” Before *name victim* can run away, *name bully* gives *him/her* a push and shouts: “Go cry a little harder!”.”

Then, the vignette stated that the teacher of their new class was supervising the playground and had seen everything that happened. Next, the teacher’s response to the incident was presented. In the Non-response Condition, it was stated that the teacher did not respond to what happened. The teacher approached another group of students and asked them which game they were playing. In the active response conditions, the teacher either called *name victim* and comforted *him/her* (Comforting the Victim) or called *name bully* and made it clear to *him/her* that bullying is not allowed (Correcting the Bully). The teacher said that there will soon be a conversation in which will be decided what the bullying student must do to make up with *name victim*. In the last condition, the teacher first comforted the victim and then corrected the bully as in the Comforting the Victim and Correcting the Bully conditions, respectively.

### Measures

#### Students’ Social Cognitions

##### Perceived Teacher Bullying Attitudes

Students’ ideas of the hypothetical teacher’s bullying attitudes were measured by two validated questionnaires that were adapted to fit with the purpose of this study. First, the perceived general attitude of the hypothetical teacher toward bullying was measured by applying the item from Saarento et al. (2013) to the story: “What do you think the teacher in the story thinks of bullying?” Students responded on a five-point scale (1: “good” to 5: “totally wrong”). Support for the construct validity of the original item has been provided by previous studies (e.g., Saarento et al., 2015a). Second, the validated attitude questionnaire of Salmivalli and Voeten (2004) was adapted to measure students’ ideas of the hypothetical teacher’s bullying attitudes instead of students’ own bullying attitudes. Prior to data collection, three items were omitted as they fitted less with the professional role of teachers (i.e., “Bullying may be fun sometimes.”; “Bullying is stupid.”; “It is funny when someone ridicules a classmate over and over again.”). The revised scale consisted of seven items (two reverse coded) measured on a four-point scale (1: “not true” to 4: “true”; e.g., “The teacher from the story thinks that joining in bullying is a wrong thing to do.”; “The teacher from the story thinks that bullying makes the victim feel bad.”). A confirmatory factor analysis (CFA) supported the one-factor structure of the scale. Following current guidelines (Hu and Bentler, 1999; Kline, 2005; Weston and Gore, 2006), model fit was evaluated by the Chi-square test, Root Mean Square Error of Approximation (RMSEA) (including the 90% CI), the Standardized Root Mean Square Residual (SRMR), the Comparative Fit Index (CFI), and the Tucker-Lewis index (TLI). Indices showed good model fit [*χ*^2^(14) = 51.32, *p* < 0.01; RMSEA = 0.07, RMSEA 90% CI = (0.05–0.08), SRMR = 0.03, CFI = 0.97; TLI = 0.95]. Standardized loadings ranged from 0.44 to 0.85. Cronbach’s alpha was 0.88. For each student, an average score was calculated. Higher scores reflect stronger perceived anti-bullying attitudes in the teacher.

##### Perceived Teacher Moral Disengagement Regarding Bullying

Students’ ideas of the hypothetical teacher’s moral disengagement regarding bullying were measured by adapting the questionnaire of Thornberg and Jungert (2013) to make students report about the hypothetical teacher instead of about themselves. The scale consisted of six items (e.g., “Bullying is okay in certain cases.”; “Bullying is not so bad… something you have to put up with.”) and measured to what extent students perceived that the hypothetical teacher reasoned in ways that justify bullying, blame the victim, and undermine the seriousness of bullying (Thornberg and Jungert, 2013). Students responded on a four-point scale (1: “not true” to 4: “true”). A CFA confirmed the one-factor structure of the scale. Indices showed good model fit [*χ*^2^(9) = 31.05, *p* < 0.01; RMSEA = 0.06, RMSEA 90% CI = (0.04–0.09), SRMR = 0.02, CFI = 0.98; TLI = 0.97]. Standardized loadings ranged from 0.67 to 0.91. Cronbach’s alpha was 0.94. For each student, an average score was calculated. Higher scores reflect stronger perceived teacher moral disengagement.

##### Willingness to Report Bullying to the Teacher

Students’ willingness to report bullying to the hypothetical teacher was measured by one item developed for the purpose of this study: “When I witness bullying, I would tell the teacher from the story about this.” Students responded on four-point scale (1: “not true” to 4: “true”).

##### Expectations Regarding Participant Role Behaviors in Bullying

Students’ ideas regarding participant role behaviors in bullying in the vignette’s class were measured by six items developed for the purpose of this study. Five items were based on the participant role questionnaire of Salmivalli and Voeten (2004) [i.e., bully, reinforcer, assistant, defender, and outsider; e.g., “In the class of the story, other students will bully.”; “In the class of the story, students will stand up for *name victim* (for example, by comforting *him/her* or by telling *name bully* to stop bullying).”]. One item was added to measure victimization (i.e., “In the class of the story, other students will be bullied.”). Students responded on four-point scale (1: “not true” to 4: “true”). An exploratory factor analysis was executed to explore whether the pro-bullying items (i.e., bully, reinforcer, and assistant) could be taken together. Factor loadings were high (0.70, 0.73, and 0.86, respectively). Additionally, the intercorrelations ranged from 0.51 to 0.63 and Cronbach’s alpha was 0.80. Thus, based on students’ scores on the bully, reinforcer, and assistant role, for each student, an average score representing their expectations regarding pro-bullying behavior was calculated. Higher scores reflect higher expectations regarding the participant role behaviors (i.e., pro-bullying behavior, defending, outsider behavior, and victimization) in the vignette’s class.

#### Students’ Own Participant Role Behaviors in Actual Bullying

Data regarding students’ own levels of different participant role behaviors in actual bullying were collected in the third wave of the longitudinal study. Peer nominations were used and five items were developed based on the participant role questionnaire of Salmivalli and Voeten (2004) (i.e., bully, reinforcer, assistant, defender, and outsider). Each item combined the descriptions of the participant role behaviors as formulated by Salmivalli and Voeten (2004) (e.g., “Which classmates bully other students at school? These are classmates who either start bullying, make others join in the bullying, always find new ways of harassing someone or do several of these actions.”). As in the study of Kärnä et al. (2010), a question to measure victimization was added: “Which classmates are bullied at school by other students?” An unlimited number of nominations was allowed (Marks et al., 2013), except for self-nominations. To ensure reliability, at least 60% of the classmates had to participate in the peer nomination procedure (Marks et al., 2013). For this reason, four classes were excluded from the analyses. Proportion scores were calculated by dividing the number of received nominations by the total number of possible nominations. An exploratory factor analysis was executed to explore whether the pro-bullying participant roles (i.e., bully, reinforcer, and assistant) could be taken together. Factor loadings were high (0.87, 0.84, and 0.82, respectively), the intercorrelations ranged from 0.67 to 0.73 and Cronbach’s alpha was 0.88. Thus, for the pro-bullying participant role behaviors, an average proportion score was calculated for each student.

#### Manipulation, Credibility, and Recognizability Checks

After completing the questionnaires measuring social cognitions and handing in the vignette, students completed three manipulation checks in a second set of questionnaires. The items measured whether students (1) perceived the situation as a bullying incident between classmates (“In the story, a student was bullied by a classmate.”, true/not true), (2) identified the correct character as the victim and bully (“In the story, *name victim* was bullied by *name bully*.”, true/not true), and (3) correctly identified the teacher response (“How did the teacher from the story respond to what had happened in the story? Choose one of the four teacher responses.”, options corresponded to the responses in the vignettes).

After completing the manipulation checks, students completed two credibility and two recognizability checks. The credibility checks measured whether students perceived the bullying incident and the teacher’s response as credible: “The bullying from the story could happen the same way in real life.”; “A real teacher could respond to bullying the same way as the teacher from the story.” Response options were “Yes, that is possible.” and “No, that is not possible” (coded as 1 and 0, respectively). The recognizability checks measured whether students were familiar with the bullying incident and the teacher’s response: “I have already seen bullying in real life that resembles the bullying from the story.”; “I have already seen the teacher’s response from the story (or a similar response) with a real teacher.” Students could respond with “yes” or “no” (coded as 1 and 0, respectively).

### Statistical Analyses

#### Preliminary Analyses

First, the distribution of students across conditions was inspected. Students (*N* = 910) were almost equally distributed across conditions (teacher response: *N*_1_ = 228, *N*_2_ = 227, *N*_3_ = 228; *N*_4_ = 227; gender of bully and victim: *N*_boys_ = 456, *N*_girls_ = 454) and Pearson’s *χ*^2^ tests revealed no significant differences between conditions regarding students’ grade [*χ*^2^(6) = 0.58, *p* = 0.997] and gender [*χ*^2^(3) = 0.63, *p* = 0.890].

Second, students’ responses to the manipulation checks were inspected. Students were excluded from all analyses when they did not answer correctly to the manipulation checks or had one or more missing values on these checks. Almost all students perceived the incident as bullying and correctly identified the victim and bully (97.4 and 95.7%, respectively). However, less students correctly identified the teacher’s response (84.4%). In particular, students confused the single responses (Comforting Victim and Correcting Bully) with the combined response (Comforting Victim and Correcting Bully). As a result, 186 of the 910 students (20.4%) were excluded from all analyses.

Third, in the remaining sample of students who were not excluded from the analyses based on their responses to the manipulation checks (*N* = 724), students who possibly provided unreliable responses were identified using two indicators. To begin with, students of which their actual teachers had indicated that they had language difficulties (*N* = 79) were identified as having possibly provided unreliable responses. Next, students who did not indicate that they read the instructions and the vignette thoroughly (*N* = 12) were identified as having possibly provided unreliable responses. As five students met both criteria, in the end, a total of 86 students (11.9%) who possibly gave unreliable responses were identified. The total sample comprised 724 students and the subsample (i.e., the total sample without students who possibly provided unreliable responses) 638 students. All further analyses were executed on the total sample and subsample. In case of similar results, only the results from the analyses on the subsample are reported. Differences in results are reported in section Sensitivity Analyses.

Fourth, the responses to the credibility and recognizability checks were inspected. Across conditions, almost all students reported that the bullying incident and teacher’s response were credible (95.9 and 90.9%, respectively). One third of the students (34.3%) were familiar with the bullying from the story and 44.4% of students were familiar with the teacher’s response. Table 1 presents the means and standard deviations across and within conditions. ANOVA indicated that students in all eight conditions (4 teacher’s response × 2 gender victim and bully) perceived the incident as equally credible and recognizable [resp. *F*(7, 637) = 1.02, *p* = 0.42; *F*(7, 635) = 1.55, *p* = 0.15]. However, significant differences between conditions appeared regarding the perceived credibility and recognizability of the teacher’s response [resp. *F*(7, 636) = 14.12, *p* < 0.001; *F*(7, 636) = 14.90, *p* < 0.001]. *Post hoc* comparisons with the Games-Howell procedure were conducted to observe which conditions significantly differed from each other regarding students’ scores on the perceived credibility and recognizability of the teacher’s response. Overall, students almost always reported significant lower credibility and recognizability scores in the non-response conditions as compared to the active response conditions (see Tables 2, 3). No other significant differences appeared.

**Table 1:** Means and SD of credibility and recognizability checks across and within conditions.

Condition	Total				Non-response				Comforting victim				Correcting bully				Comforting victim + Correcting bully			
	boys		girls		boys		girls		boys		girls		boys		girls		boys		girls	
**Variable**	*N*	*M* (*SD*)	*N*	*M* (*SD*)	*N*	*M* (*SD*)	*N*	*M* (*SD*)	*N*	*M* (*SD*)	*N*	*M* (*SD*)	*N*	*M* (*SD*)	*N*	*M* (*SD*)	*N*	*M* (*SD*)	*N*	*M* (*SD*)
Credibility of bullying incident^a^	308	0.95 (0.22)	330	0.97 (0.18)	85	0.93 (0.26)	91	0.93 (0.25)	82	0.98 (0.16)	85	0.98 (0.15)	69	0.94 (0.24)	75	0.97 (0.16)	72	0.96 (0.20)	79	0.99 (0.11)
Credibility of teacher response^b^	307	0.93 (0.26)	330	0.90 (0.30)	84	0.81 (0.40)	91	0.69 (0.46)	82	0.95 (0.22)	85	0.95 (0.21)	69	0.99 (0.12)	75	0.99 (0.12)	72	0.97 (0.17)	79	0.99 (0.11)
Recognizability of bullying incident^a^	306	0.35 (0.48)	330	0.34 (0.47)	84	0.29 (0.45)	91	0.29 (0.45)	82	0.43 (0.50)	85	0.38 (0.49)	68	0.31 (0.47)	75	0.27 (0.45)	72	0.38 (0.49)	79	0.43 (0.50)
Recognizability of teacher response^b^	307	0.50 (0.50)	330	0.40 (0.49)	85	0.16 (0.37)	91	0.15 (0.36)	82	0.57 (0.50)	85	0.47 (0.50)	68	0.69 (0.47)	75	0.49 (0.50)	72	0.61 (0.49)	79	0.51 (0.50)

a ANOVA showed no significant differences between conditions.

b ANOVA showed significant differences between conditions and *post hoc* comparisons were executed (see Tables 2, 3).

**Table 2:** *Post hoc* comparisons of credibility of the teacher’s response across conditions.

		Non-response – boys	Non-response – girls
		***M* = 0.81**	***M* = 0.69**
Comforting victim – boys	*M* = 0.95	∆*M* = −0.14, *SE* = 0.05	∆*M* = −0.26^**^, *SE* = 0.05
Comforting victim – girls	*M* = 0.95	∆*M* = −0.14, *SE* = 0.05	∆*M* = −0.26^**^, *SE* = 0.05
Correcting bully – boys	*M* = 0.99	∆*M* = −0.18^*^, *SE* = 0.05	∆*M* = −0.29^**^, *SE* = 0.05
Correcting bully – girls	*M* = 0.99	∆*M* = −0.18^*^, *SE* = 0.05	∆*M* = −0.29^**^, *SE* = 0.05
Comforting victim + Correcting bully – boys	*M* = 0.97	∆*M* = −0.16, *SE* = 0.05	∆*M* = −0.28^**^, *SE* = 0.05
Comforting victim + Correcting bully – girls	*M* = 0.99	∆*M* = −0.18^*^, *SE* = 0.05	∆*M* = −0.30^**^, *SE* = 0.05

Mean differences regarding the credibility of the teacher’s response between the non-response conditions and the active response conditions are presented. Other *post hoc* comparisons revealed no significant differences.

* *p* < 0.010

** *p* < 0.001.

**Table 3:** *Post hoc* comparisons of recognizability of the teacher’s response across conditions.

		Non-response – boys	Non-response – girls
		***M* = 0.16**	***M* = 0.15**
Comforting victim – boys	*M* = 0.57	∆*M* = −0.41^**^, *SE* = 0.07	∆*M* = −0.42^**^, *SE* = 0.07
Comforting victim – girls	*M* = 0.47	∆*M* = −0.31^**^, *SE* = 0.07	∆*M* = −0.32^**^, *SE* = 0.07
Correcting bully – boys	*M* = 0.69	∆*M* = −0.53^**^, *SE* = 0.07	∆*M* = −0.54^**^, *SE* = 0.07
Correcting bully – girls	*M* = 0.49	∆*M* = −0.33^**^, *SE* = 0.07	∆*M* = −0.34^**^, *SE* = 0.07
Comforting victim + Correcting bully – boys	*M* = 0.61	∆*M* = −0.45^**^, *SE* = 0.07	∆*M* = −0.46^**^, *SE* = 0.07
Comforting victim + Correcting bully – girls	*M* = 0.51	∆*M* = −0.34^**^, *SE* = 0.07	∆*M* = −0.35^**^, *SE* = 0.07

Mean differences regarding the recognizability of the teacher’s response between the non-response conditions and the active response conditions are presented. Other *post hoc* comparisons revealed no significant differences.

** *p* < 0.001.

#### Main Analyses

IBM SPSS Statistics 26 was used to examine the effects of students’ perceptions of teacher responses to bullying on their social cognitions. Mplus 8 (Muthén and Muthén, 1998-2017) was used to conduct the factor analyses (see section Measures). In the CFA’s, maximum likelihood estimation with robust standard errors (MLR estimator) combined with the “complex analysis” feature was used to deal with item-level missingness, non-normality and non-independence of the observations (Muthén and Muthén, 1998-2017; Newman, 2014). Mplus 8 was also used to estimate the intraclass correlations (ICC’s) and design effects at the level of the classroom. These statistics showed that for all outcomes little variance was explained by class (range ICC’s: <0.01–0.02) and that the effects of dependence on standard error estimates were small (range design effects: 1.00–1.25; Peugh, 2010). Therefore, multilevel modeling was not needed (Peugh, 2010) and analysis of covariance (ANCOVA) could be used.

In step 1 of the analyses, the effects of relevant background variables (i.e., students’ age and gender) and gender of bully and victim on the outcomes were examined. Variables that significantly predicted the respective outcomes were added as control variables to the models of these outcomes in steps 2 and 3. In step 2, the effect of teacher responses on the outcomes was examined, while controlling for the identified control variables. If the effect of teacher responses was significant, either planned or *post hoc* comparisons were executed depending on whether or not hypotheses had been formulated prior to the analyses. Regarding the planned comparisons, bootstrapping was used and the results of the contrasts not assuming equal variances were inspected (Field, 2017). For the effect size, Cohen’s *d* for unequal-n design was calculated (Rosnow et al., 2000) with *d* = 0.20, 0.50, and 0.80 representing a small, medium, and large effect, respectively. Regarding the *post hoc* comparisons, the Games-Howell procedure with significance level 0.01 was used as the sample sizes and the variances were not equal in the different conditions. In step 3, the interactions between teacher responses and students’ own levels of participant role behaviors in bullying were examined, while controlling for their main effects and the identified control variables. To control for multiple testing in the ANCOVA and planned comparison analyses, Bonferroni correction was used (Field, 2017). As 26 tests were carried out (step 2: 8 ANCOVA, 10 planned comparisons; step 3: 8 ANCOVA), the correction resulted in an alpha of 0.002.

All analyses were executed on the total sample and the subsample (i.e., without students who possibly provided unreliable responses). As the results regarding the validity and reliability of the measures, the credibility and recognizability checks and the descriptives were similar, only the results of the analyses on the subsample are reported. However, some differences in results concerning the main analyses appeared and are reported (see section Sensitivity Analyses). Also, as heterogeneity of variance could have affected the results, parameter estimates with robust standard errors (HC4) were inspected for the analyses on the subsample and the total sample (Field, 2017). All significant effects remained significant when robust standard errors were used.

## Results

Table 4 displays the descriptive statistics of the dependent variables, Across and within conditions. Extreme outliers (| *z* | > 3.29) within conditions were identified (see Table 4; Field, 2017). As the amount of extreme outliers was limited, they were not excluded from the analyses.

**Table 4:** Descriptive statistics of dependent variables across and within conditions.

Condition	Total		Non-response		Comforting victim		Correcting bully		Comforting victim + Correcting bully	
Variable	*N*	*M* (*SD*)	*N* (*Out*.)	*M* (*SD*)	*N* (*Out*.)	*M* (*SD*)	*N* (*Out*.)	*M* (*SD*)	*N* (*Out*.)	*M* (*SD*)
General attitude of teacher	614	3.89 (1.25)	171 (4)	2.16^a^ (0.64)	161 (1)	4.19^b,c^ (0.75)	135	4.77^b,d^ (0.42)	147 (1)	4.77^b,d^ (0.44)
Attitudes of teacher	638	3.22 (0.76)	176	2.27^a^ (0.63)	167 (2)	3.52^b^ (0.44)	144 (1)	3.59^b^ (0.41)	151 (2)	3.65^b^ (0.37)
Moral disengagement of teacher	637	1.59 (0.80)	175	2.71^a^ (0.64)	167 (1)	1.26^b,c^ (0.34)	144 (4)	1.12^b,d^ (0.20)	151 (4)	1.12^b,d^ (0.22)
Willingness to report bullying	636	3.68 (0.64)	175 (4)	3.69 (0.66)	166	3.52^a^ (0.80)	144	3.74^b^ (0.53)	151 (3)	3.79^b^ (0.46)
Pro-bullying behaviors	638	1.90 (0.77)	176	2.43^a^ (0.77)	167	1.85^b,c^ (0.73)	144 (1)	1.65^b^ (0.64)	151 (1)	1.58^b,d^ (0.56)
Defending	637	2.97 (0.92)	176	2.62^a^ (0.95)	167	3.02^b^ (0.85)	144	3.06^b^ (0.93)	150	3.21^b^ (0.85)
Victimization	634	1.99 (0.93)	175	2.39^a^ (1.03)	165	1.99^b^ (0.87)	144	1.74^b^ (0.82)	150	1.75^b^ (0.82)
Outsider behaviors	635	2.44 (0.93)	175	2.39 (0.96)	166	2.43 (0.90)	143	2.47 (0.98)	151	2.48 (0.87)

For each outcome, superscript lowercase letters a, b, c, d, show which means significantly differ from each other based on planned or *post hoc* comparisons. 1 = perceived general attitude toward bullying of the hypothetical teacher; 2 = perceived attitudes toward bullying of the hypothetical teacher; 3 = perceived moral disengagement regarding bullying of the hypothetical teacher; 4 = willingness to report bullying to the hypothetical teacher; 5 = expectations regarding pro-bullying behaviors in the vignette’s class; 6 = expectations regarding defending in the vignette’s class; 7 = expectations regarding victimization in the vignette’s class; and 8 = expectations regarding outsider behaviors in the vignette’s class; Out, outliers.

### Control Variables

ANOVA revealed that, in general, girls were more willing to report bullying to the hypothetical teacher [*F*(1, 631) = 7.01, *p* = 0.008; *M*_girls_ = 3.74, *SE* = 0.56, *M*_boys_ = 3.61, *SE* = 0.71]. Also, girls generally expected more outsider behaviors in the vignette’s classroom [*F*(1, 630) = 8.58, *p* = 0.004; *M*_girls_ = 2.54, *SE* = 0.93, *M*_boys_ = 2.32, *SE* = 0.91]. Boys, on the other hand, expected more pro-bullying behaviors and victimization in the vignette’s classroom [respectively, *F*(1, 633) = 5.83, *p* = 0.016; *M*_girls_ = 1.83, *SE* = 0.74, *M*_boys_ = 1.98, *SE* = 0.79; *F*(1, 629) = 4.77, *p* = 0.029; *M*_girls_ = 1.91, *SE* = 0.90, *M*_boys_ = 2.08, *SE* = 0.97]. Further, with increasing age, more pro-bullying behaviors and less defending were expected in the vignette’s classroom [*F*(1, 633) = 14.14, *p* < 0.001; *F*(1, 632) = 6.64, *p* = 0.01]. Finally, more defending was expected in the vignette’s classroom when the hypothetical bully and victim were girls [*F*(1, 632) = 4.38, *p* = 0.037; *M*_hypo.girls_ = 2.90, *SE* = 0.94, *M*_hypo.boys_ = 3.04, *SE* = 0.90]. No other significant effects at alpha ≤0.05 were observed regarding the control variables. The variables that were found to significantly predict the respective outcomes were included in further analyses of these outcomes as a control variable.

### Effects of Teacher Responses

First, *perceived teacher bullying attitudes* and *perceived teacher moral disengagement* were significantly affected by the teacher’s response to bullying [general attitude: *F*(3, 610) = 712.05, *p* < 0.001, *η_p_*^2^ = 0.78; attitudes: *F*(3, 634) = 321.73, *p* < 0.001, *η_p_*^2^ = 0.60; moral disengagement: *F*(3, 633) = 625.54, *p* < 0.001, *η_p_*^2^ = 0.75]. Planned comparisons revealed that weaker anti-bullying attitudes and more moral disengagement were perceived for the teacher in the Non-response Condition compared to the active response conditions [either Correcting the Bully, Comforting the Victim, or a combination of Correcting the Bully and Comforting the Victim; general attitude: *t*(270.89) = 43.52, *p* = 0.001, *d* = 4.17; attitudes: *t*(233.22) = 25.84, *p* = 0.001, *d* = 2.68; moral disengagement: *t*(195.84) = −31.19, *p* = 0.001, *d* = −3.53]. The sizes of these effects were very large. Further, planned comparisons revealed that the teacher’s general attitude toward bullying was perceived to be less negative in the Comforting the Victim Condition compared to the Correcting the Bully Conditions [with and without Comforting the Victim; *t*(221.15) = 9.03, *p* = 0.001, *d* = 1.03]. Additionally, more moral disengagement was perceived in the Comforting the Victim Condition compared to the Correcting the Bully Conditions [with and without Comforting the Victim; *t*(238.82) = −4.72, *p* = 0.001, *d* = −0.52]. These effects were large and medium in size. No significant differences regarding perceived teacher bullying attitudes and perceived teacher moral disengagement were observed between only Correcting the Bully and the combined response of Correcting the Bully and Comforting the Victim.

Second, *students’ willingness to report bullying to the teacher* was significantly affected by the teacher’s response [*F*(3, 631) = 5.30, *p* = 0.001, *η_p_*^2^ = 0.03]. Planned comparisons showed no significant differences between the Non-response Condition and the active response conditions (either Correcting, Comforting or Both). However, in the Correcting the Bully Conditions (with and without Comforting the Victim), students were more willing to report bullying to the teacher compared to students in the Comforting the Victim Condition [*t*(237.39) = 3.48, *p* = 0.002, *d* = 0.38]. This effect was small in size. No significant difference was observed between only Correcting the Bully and the combined response of Correcting the Bully and Comforting the Victim.

Third, *students’ expectations regarding pro-bullying behaviors*, *defending* and *victimization* in the vignette’s class were predicted by the teacher’s response [pro-bullying: *F*(3, 631) = 53.24, *p* < 0.001, *η_p_*^2^ = 0.20; defending: *F*(3, 630) = 12.56, *p* < 0.001, *η_p_*^2^ = 0.06; victimization: *F*(3, 629) = 19.94, *p* < 0.001, *η_p_*^2^ = 0.09]. *Students’ expectations regarding outsider behaviors* in the vignette’s classroom were not significantly predicted by the teacher’s response. Planned comparison revealed that less pro-bullying behaviors, more defending and less victimization were expected in the active response conditions compared to the Non-response Condition [pro-bullying: *t*(273.24) = −11.24, *p* = 0.001, *d* = −1.08; defending: *t*(294.22) = 5.72, *p* = 0.001, *d* = 0.53; victimization: *t*(266.57) = −6.56, *p* = 0.001, *d* = −0.64]. These effects were medium to large. Further, *post hoc* comparisons with the Games-Howell procedure revealed that less pro-bullying behaviors were expected in the condition where the teacher both Comforted the Victim and Corrected the Bully compared to the condition in which only the Victim was Comforted [∆*M* = −0.27, *SE* = 0.07, *p* = 0.002, 99% CI = (−0.49, −0.04)]. Regarding *students’ expectations for defending* and *victimization*, *post hoc* comparisons with the Games-Howell procedure revealed no significant differences between the active responses.

No interaction effects between the teacher’s response and students’ own participant role behaviors in bullying were significant at the Bonferonni corrected alpha level (*α* = 0.002).

### Sensitivity Analyses

When the results based on the total sample were compared with the results based on the subsample, two differences were observed. First, in the total sample, the gender of the bully and victim did not significantly affect students’ expectations regarding defending in the vignette’s classroom [*F*(1, 716) = 3.82, *p* = 0.051; *M*_hypo.girls_ = 2.88, *SE* = 0.95, *M*_hypo.boys_ = 3.00, *SE* = 0.95]. Second, in the total sample, students did not expect significantly (at alpha 0.002) less pro-bullying behaviors in the condition where the teacher both Comforted the Victim and Corrected the Bully compared to the condition in which only the Victim was Comforted [∆*M* = −0.25, *SE* = 0.07, *p* = 0.003, 99% CI = (−0.47, −0.03)].

## Discussion

Bullying affects students worldwide and is associated with a wide range of short- and long-term difficulties, especially for victims (Arseneault, 2018). As bullying is often embedded in classrooms, teachers are considered to be key figures in bullying intervention (Brendgen and Troop-Gordon, 2015). A limited number of studies have shown that by their responses to bullying incidents, teachers can affect bullying levels in their classrooms (e.g., van der Zanden et al., 2015; Campaert et al., 2017). To get further insight in how teacher responses predict bullying, scholars have pointed to different student social cognitions related with bullying processes that could be impacted by teacher responses (Troop-Gordon and Quenette, 2010; Menesini, 2019). However, thus far, only a few studies, of which most are correlational, have examined effects of teacher responses on student cognitions. This study aimed to fill this gap and to provide insight in whether and how teacher responses to bullying influence students’ cognitions, which, in turn, can be assumed to explain students’ bullying-related behaviors. Experimental vignettes were used to investigate the effects of four teacher responses to a same bullying incident (Non-response, Comforting Victim, Correcting Bully, and a combination of Comforting Victim and Correcting Bully) on four student social cognitions: (1) perceived teacher attitudes toward bullying, (2) perceived teacher moral disengagement regarding bullying, (3) student willingness to report bullying to the teacher, and (4) expectations regarding bullying participant role behaviors in the vignette’s classroom.

Based on socio-cognitive theory (Bandura, 1986), students were expected to cognitively process teacher responses to bullying and to take different cues from them. First, students were expected to make interpretations regarding the *teacher’s attitudes toward bullying*. Building on theory and previous research (Veenstra et al., 2014; van der Zanden et al., 2015; Demol et al., in revision), it was hypothesized that students would perceive stronger anti-bullying attitudes when the hypothetical teacher actively responded to the bullying. Further, it was hypothesized that even stronger anti-bullying attitudes would be perceived when the teacher corrected the bully. The results confirmed the hypotheses and provided further support for previous findings. Our findings suggest that when teachers actively respond to bullying and especially when they confront bullies, students perceive stronger teacher anti-bullying attitudes. Conversely, when teachers do not respond, students may get the impression that teachers condone bullying or that they do not care about it. Based on social learning theory (Bandura, 1977), perceived teacher attitudes can be assumed to inform students about teachers’ expectations regarding acceptable behavior in class which may predict students’ own behavior. This is in line with previous research showing that students’ perceptions of teachers’ bullying attitudes were longitudinally related with bullying levels (Saarento et al., 2015a) and concurrently with victimization levels (Saarento et al., 2013; Cortes and Kochenderfer-Ladd, 2014). Additionally, weaker teacher anti-bullying attitudes as perceived by victims might be associated with more victimization as victims are less inclined to seek help from these teachers (Blomqvist et al., 2020).

A second cue that students were expected to take concerned *teacher’s moral disengagement regarding bullying*. No previous studies were available, but based on the expectations regarding perceived teacher attitudes, students were expected to perceive less moral disengagement in the hypothetical teacher who actively responded. This effect was also expected to be stronger when the bully was corrected. These hypotheses were confirmed and complement the findings of Campaert et al. (2017) who showed that teacher responses are predictive for students’ own levels of moral disengagement in bullying. Based on social learning theory (Bandura, 1977), it can be expected that when students perceive from teacher responses that teachers justify bullying, blame victims, or undermine the seriousness of bullying, students could reason about bullying the same way. This is problematic as research has shown students’ moral disengagement regarding bullying is significantly related with aggressive behavior (Gini et al., 2014). Instead, when students understand moral principles and know that there is no valid justification for bullying, they can be protected from bullying (Zych et al., 2017). Therefore, teachers should model behavior that promotes social, emotional, and moral competencies in students (Zych et al., 2017).

Third, based on previous research (e.g., Aceves et al., 2010; Blomqvist et al., 2020), students’ willingness to report bullying to the hypothetical teacher was expected to be higher when the teacher actively responded to the bullying. This effect was also expected to be stronger when the teacher corrected the bully (Veenstra et al., 2014; Blomqvist et al., 2020). We found that students’ willingness to report bullying to the teacher was high in all four conditions. It seems that most students, as bystanders, considered it as their duty to report bullying to teachers, regardless of the teacher’s response in the vignette. It is a positive finding that students’ telling about bullying was high as this has been found to be an important predictor of teacher involvement in bullying (Novick and Isaacs, 2010) and as classrooms with higher willingness to report bullying have lower levels of victimization (Cortes and Kochenderfer-Ladd, 2014). However, students’ responses to this outcome might have been influenced by social desirability as the social norm is to stand up in case of aggression. Although, unexpectedly, willingness to report bullying was not higher when the teacher actively responded compared to non-response, differences between the active responses confirmed that students would be more willing to report bullying when the teacher corrected the bully than when only the victim was comforted. When teachers confront bullies, they explicitly show that they try to stop bullying (Veenstra et al., 2014) and that might be key for students willing to report. However, it is important to consider that bystanders’ intentions to report bullying were investigated. Findings could be different for victims. For instance, fear of retaliation, which could be particularly pronounced in victims, could predict less willingness to report bullying to teachers especially when they direct responses to bullies (Yoon and Barton, 2008).

The fourth and last investigated cognition was *students’ expectations regarding participant role behaviors in bullying in the vignette’s class*. Based on previous research, Yoon and Bauman (2014) argued that by their responses to bullying, teachers can set expectations for potential bullies, victims, and other students’ future behaviors and the relational climate in class. Therefore, it was hypothesized that when the teacher actively responded, students would expect less pro-bullying behaviors, more defending, less victimization, and less outsider behaviors. Due to the unavailability of previous research, differences between the active responses were explored. In line with the hypotheses, students expected less pro-bullying behaviors, more defending and less victimization in the classroom when the teacher actively responded. For outsider behaviors, there were no significant differences between any responses. Further, there were no significant differences between the active responses regarding expectations for defending and victimization. Less pro-bullying behaviors were expected when the teacher both comforted the victim and corrected the bully vs. when only the victim was comforted, but this difference was not significant in the analyses on the total sample. Together these findings indicate that students do think that active teacher responses have beneficial effects on bullying processes in classrooms. Students do expect less bullying, more defending and less victimization following active teacher responses. This is in line with the findings from Campaert et al. (2017) showing that non-response predicted higher bullying and victimization, and disciplinary sanctions and victim support predicted less bullying and victimization respectively.

Finally, it was explored whether students’ own levels of different participant role behaviors moderated the effect of teacher responses on their cognitions. Due to the unavailability of previous research, it was only tentatively expected that for relatively higher levels of victimization and defending, the effect of teacher response would be stronger. For relatively higher levels of pro-bullying behaviors (i.e., bullying, assisting, and reinforcing), the effect of teacher response was expected to be less strong. These expectations were not confirmed. Although, in this study, no moderating effects of students’ own bullying participant role behaviors on their cognitions were found, it is possible that students’ cognitions about actual teacher responses are affected by individual characteristics such as their bullying-related behaviors and beliefs.

To conclude, first, the results of the current study are in line with social cognitive (Bandura, 1986) and goal-framing theory (Veenstra et al., 2014) and confirm findings by Demol et al. (in revision) showing that students can deduce beliefs from an imaginary teacher’s behavior. Second, in the current study, non-response was also found to have important effects on students’ beliefs indicating that it should actually be considered as a response. Our findings indicate that students believe that a non-responding teacher thinks less negatively about bullying and justifies it more. In addition, our study suggests that students believe that non-response, compared to active responses, would have adverse effects on bullying processes in the classroom (i.e., more bullying and victimization, less defending). Third, a confronting response directed to the bully (correcting) had a stronger effect on students’ cognitions than a supporting response directed to the victim (comforting). Students perceived even stronger anti-bullying attitudes and even less moral disengagement when the teacher corrected the bully. Further, when the teacher corrected the bully, students were more willing to report bullying to the teacher. Overall, this study found clear evidence for effects of teacher responses on student perceptions about the teacher and about classroom dynamics. However, evidence regarding effects on students themselves, more specifically on their willingness to report bullying, was less clear.

### Strengths, Limitations, and Suggestions for Future Research

The current study used experimental vignettes to investigate whether and how teacher responses to bullying influence different bullying-related student cognitions in a large sample of upper elementary school students. The design, including the use of a vignette, permitted to manipulate teacher responses without interfering in reality and to draw causal conclusions. The statistical analyses took into account several characteristics of the data (e.g., Cohen’s *d* for unequal-n design, *post hoc* comparisons with Games-Howell procedure), controlled for multiple testing and sensitivity analyses largely confirmed the findings. However, the study also has a number of limitations that are worth noticing. First, as hypothetical stories were used, students’ cognitions should also be interpreted as hypothetical reactions. It is not certain that students’ cognitions following actual bullying and actual teacher responses would be similar. This study included credibility and recognizability checks to verify whether the bullying incident and teacher’s response were credible and recognizable for students. If so, the likelihood that the cognitions would reflect real life cognitions would be higher. Almost all students perceived the bullying incident as credible, but the teacher’s non-response was perceived to be less credible than the active responses. Additionally, non-response was less recognizable to the students. As a result, students’ cognitions following non-response could be less realistic. Although the use of experimental vignettes has several merits, observational studies, preferably longitudinal, are needed to confirm findings in actual classrooms.

Second, as students were randomly assigned to conditions, the risks that individual- and class-level factors influenced the findings were reduced. However, in future research, it could be interesting to investigate main and moderating effects of these factors on students’ cognitions (e.g., students’ own attitudes toward bullying, classroom bullying levels, actual teacher’s responses to bullying).

Third, although the investigated teacher responses were carefully selected based on previous studies, the study is limited in that only four different immediate teacher responses were investigated. The results of this study should be interpreted in relation to these specific responses and should not be generalized to other responses. Future studies could focus on other responses (e.g., involving others, mediation) or investigate responses in a more differentiated way (e.g., different responses targeting the bully; see Garandeau et al., 2016).

### Practical Implications

First, it is important for teachers to realize that students can deduce beliefs from their observations of teacher responses to bullying. These beliefs or student cognitions, in turn, can affect bullying processes in the classroom for better or worse. For instance, when students perceive from teachers’ non-response that their teachers do not disapprove of bullying, they can be more likely to engage in bullying (Saarento et al., 2013).

Second, the present findings further emphasize the importance of active teacher responses to bullying and especially responses that explicitly show that bullying is not tolerated. Teacher responses are an important part of bullying intervention (Yoon and Bauman, 2014). However, previous research has indicated that sometimes teachers do not intervene and that they are often ill-prepared to effectively deal with bullying (Yoon and Bauman, 2014). Teacher training could focus on several aspects that have been found to increase chances of teacher intervention such as recognizing bullying situations and teacher’s self-efficacy beliefs (for an overview, see Newman et al., 2010).

## Data Availability Statement

The raw data supporting the conclusions of this article will be made available by the authors, without undue reservation.

## Ethics Statement

The studies involving human participants were reviewed and approved by The Social and Societal Ethics Committee (SMEC) of KU Leuven. Written informed consent to participate in this study was provided by the participants’ legal guardian/next of kin.

## Author Contributions

KD conceived of the study, coordinated the data collection, constructed the hypotheses, performed the statistical analyses, interpreted the results, and drafted the manuscript. KV conceived of the study, constructed the hypotheses, interpreted the results, and drafted the manuscript. CS conceived of the study, constructed the hypotheses, interpreted the results, and drafted the manuscript. HC conceived of the study, supervised the data collection, constructed the hypotheses, interpreted the results, and drafted the manuscript. All authors contributed to the article and approved the submitted version.

### Conflict of Interest

The authors declare that the research was conducted in the absence of any commercial or financial relationships that could be construed as a potential conflict of interest.
